# Imaging in the Diagnosis and Monitoring of Children with Idiopathic Scoliosis

**DOI:** 10.2174/1874325001711011500

**Published:** 2017-12-29

**Authors:** Shu-Yan Ng, Josette Bettany-Saltikov

**Affiliations:** Wanchai Chiropractic Clinic, 11/fl China Hong Kong Tower, 8 Hennessy Road, Wanchai, Hong Kong

**Keywords:** Adolescent idiopathic scoliosis, Imaging, Radiography, EOS^®^, 3D ultrasound imaging, Surface topography, Computed tomography, Magnetic resonance imaging

## Abstract

The paper reviews the current imaging methods in the diagnosis and monitoring of patients with adolescent idiopathic scoliosis. Radiography is generally used in the initial diagnosis of the condition. Postero-anterior erect full spine radiograph is generally prescribed, and is supplemented by lateral full spine radiograph when indicated. To reduce the radiation hazard, only the area of interest should be exposed, and follow-up radiographs should be taken with as few projections as possible. When available, EOS^®^ stereoradiography should be used. The radiation of the microdose protocol is 45 times less than that of the conventional radiography. Surface topography offers another approach to monitoring changes of curvatures in AIS patients. Recently, 3D ultrasound has been found to be able to measure the Cobb angle accurately. Yet, it is still in the early developmental stages. The inherent intrinsic and external limitations of the imaging system need to be resolved before it can be widely used clinically. For AIS patients with atypical presentation, computed tomography (CT) and/or magnetic resonance imaging (MRI) may be required to assess for any underlying pathology. As CT is associated with a high radiation dose, it is playing a diminishing role in the management of scoliosis, and is replaced by MRI, which is also used for pre-operative planning of scoliosis.

The different imaging methods have their limitations. The EOS^®^ stereoradiography is expensive and is not commonly available. The surface topography does not enable measurement of Cobb angle, particularly when the patient is in-brace. The 3D ultrasound scanning has inherent intrinsic technical limitation and cannot be used in all subjects. Radiography, however, enables diagnosis and monitoring of the adolescent idiopathic scoliosis (AIS). It is thus the gold standard in the evaluation and management of scoliosis curves.

## 1 INTRODUCTION

Adolescent idiopathic scoliosis afflicts 2-3% of the population. It is generally noticed by parents, friends or detected during a spinal screening utilizing a scoliometer which measures the angle of trunk rotation (ATR). Various studies have shown that the method is reliable, with low inter-observer and intra-observer error [1]. Generally, when the ATR is ≥ 5^o^, the patient is likely to have a lateral spinal curvature in excess of 10^o^. When the ATR is ≥ 7^o^, the patient needs to be referred for radiographs for evaluation [2].

Different imaging methods are used in the management of idiopathic scoliosis. These include conventional radiography, the low-dose stereoradiography (EOS^®^), surface topography, 3D ultrasound, computed tomography (CT) and magnetic resonance imaging (MRI). Fluoroscopy is generally used intra-operatively and is not discussed in this review paper.

These various imaging methods have different indications, advantages and disadvantages. At present, there is no fixed algorithm as to when each imaging method is indicated [3] and radiography is still the gold standard in the diagnosis, evaluation and management of spinal deformities during growth. The different imaging methods are discussed below.

## 2 RADIOGRAPHY

Radiography is commonly used in the diagnosis and follow-up management of patients with scoliosis. Depending on the requirements of the physician, the standing frontal and lateral views of the full spine are generally requested [4. 5]. The general recommendation is to have the film covering the area of the back from C7 to the sacrum and include both iliac crests to enable evaluation of the Risser index [5, 6]. In young patients, the radiograph may need to include the hip to determine if the tri-radiate cartilage, which is the Y-shape epiphyseal plate between the ilium, ischium and pubis has fused [7], as closure of the cartilage occurs after the peak height growth velocity phase [8].Weiss and Seibel (2013), however, are of the opinion that it is not necessary to include the entire pelvis and the hip, as this increases the radiation exposure and does not provide useful information in the management of scoliosis [9]. Instead, their suggestion is to limit the x-ray exposure of the back both from T1 to S3 as well as to cover the medial aspect of the iliac crest. Exclusion of the lateral pelvis does not permit the identification of a Risser stage less than 3. This, however, does not have any impact clinically in the management of AIS as patients with a Risser sign of 0 can be identified through observation of the Tanner stage, which is a scale depicting the physical development of children, adolescents and adults. Patients in Risser stage 0 are generally in Tanner stage 1 [10]. In Tanner stage 1, the secondary sexual characteristics have yet to develop. The inclusion of the medial aspect of the iliac crest in a PA film permits the identification of Risser stages 3 and 4. This also provides sufficient information to enable practitioners who are treating patients with braces as to when they can start weaning patients off the brace [9].

The lateral film should cover the area from T1 to the sacrum and be used as a reference for future comparison. When the sagittal profile is not markedly abnormal, lateral spinal films can be dispensed [9] to reduce the radiation dosage to the patient.

These films while termed as frontal and lateral view are not the frontal and lateral view of the spinal deformity. The x-rays are actually the oblique view of the spinal deformity. Special views are generally required to ascertain the “true” frontal and lateral view of the scoliotic deformity.

### 2.1 Diagnosis and Evaluation

During the initial evaluation, plain radiographs should be used for diagnosis. In the presence of atypical scoliosis and segmental defects, the patient should be referred for MRI examination, as the incidence of spinal cord abnormalities is high in these patients [11-15]. Further, in the absence of an increased spinal canal diameter or other signs that are indicative of secondary scoliosis or syndromic scoliosis such as Marfan syndrome and neurofibromatosis, the patient can be diagnosed with idiopathic scoliosis.

In the PA radiograph, the curve type, Cobb angle, apical vertebral rotation (AVR), coronal balance and Risser staging need to be measured and determined. Clinicians should also record the upper and lower end vertebra used for measuring, the apex and whether or not vertebral wedging is present.

### 2.2 Cobb Angle

As previously stated, scoliosis is a 3D spinal deformity. Despite this being the case, the evolution of curves together with the success or failure of scoliosis treatment is still to a large extent and in the large majority of clinics worldwide determined by changes in the Cobb angle, which is a 2D measurement [16]. Not surprisingly, this measurement is subject to error. In 1993, Beauchamp *et al.* also reported that the Cobb angle is subject to diurnal changes. Indeed, the mean Cobb angle in the afternoon can be 5^o^ more than that measured in the morning [17]. When the vertebral end points were not defined, the mean inter-observer and intra-observer variation were relatively high, at 7.2^o^ and 4.9^o^, respectively [18]. When the end points were defined, however, the variability reduced, suggesting the importance of selecting consistent end points when evaluating serial radiographs. At present, the margin of error of Cobb angle measurement is generally regarded to be 5^o^. Morrissy *et al.* (1990) regards a 10^o^ change in Cobb angle necessary to be 95% confident that there is indeed a change of scoliosis [18].

According to the Scoliosis Research Society (SRS) 2005 and SOSORT 2006 and 2011 guidelines, patients with Risser staging from 0 – 2 and Cobb angle between 25-40^o^ need to be treated with bracing [16, 19, 20]. Bracing should preferably be done in conjunction with physical rehabilitation (scoliosis-specific exercises), as these curve-specific exercises have not only been found to reduce curve progression [21, 22] but they also stabilize the curve during brace weaning [20]. An adolescent patient with a Cobb angle ≥ 45^o^ and remaining spinal growth should be referred for surgery [16, 20]. Recently, however, Bettany-Saltikov *et al.* (2015) identified no evidence to support the effectiveness of surgical intervention compared to non-surgical interventions for patients with AIS [23].

### 2.3 Apical Vertebral Rotation

**Fig. (1):**
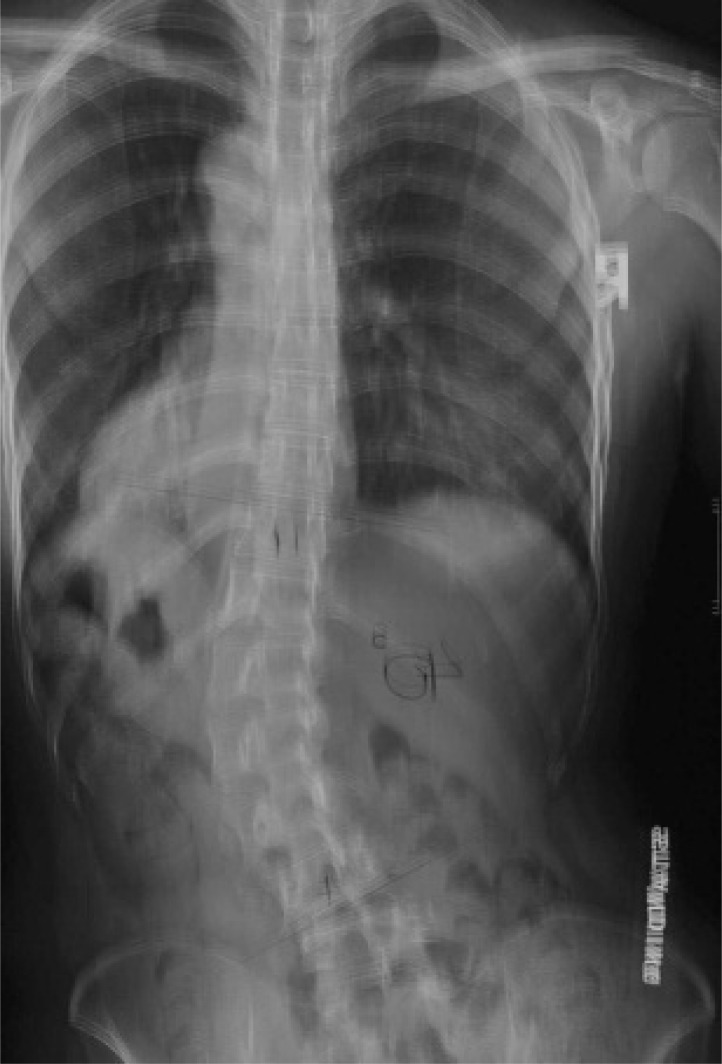
Rotation of vertebral body and spinous process in scoliosis. From the x-ray, it is apparent that the vertebral body of L1 (the apex of the curve) rotates towards the convexity of the curve, which is the left and the spinous process rotates to the concavity of the curve.

**Fig. (2):**
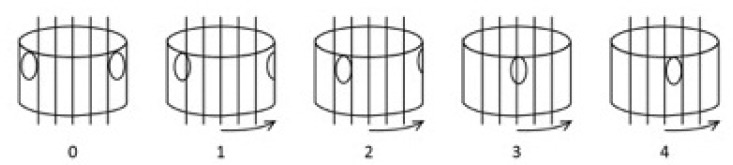
The Nash and Moe method of determining vertebral rotation clinically. The apical vertebral body is divided into six equal segments longitudinally. When both pedicles are in view, there is no vertebral rotation. It is graded as “0”. When the pedicle in the concave side (the right side) starts disappearing, it is graded as “1”. When the pedicle disappears, it is graded as “2”. When the contralateral pedicle (pedicle in the convex side) is in the midline of the vertebra, it is graded as “3”. When it crosses the midline of the vertebra, it is graded as “4”.

At the apex of a scoliotic curve, the vertebral body rotates towards the convexity of the curve and the spinous process towards the concavity of the curve (Fig. **1**). The extent of apical vertebral rotation is generally determined from the PA spinal film using a number of different methods. These include the Nash and Moe method [24], the Raimondi [25] or the Perdriolle methods and the Vidal method [25]. The Nash and Moe method is a radiological method. In this method, the vertebra is divided into six segments longitudinally and grades 0 to 4 are assigned, depending on the location of pedicle within segments (Fig. **2**). When there is no vertebral rotation in the spine and the pedicles are symmetrical, it is graded as 0. When the convex pedicle rotates past the midline, it is regarded as Grade 4. This method has some drawbacks, however, as it only provides a rough approximation of axial rotation [26]. In 1993, Ho *et al.* conducted a research study which found that CT scans showed a rotation of up to 11 degrees whilst the Nash and Moe method showed that there was no vertebral rotation at all (0 grade) [24]. Further, this method does not consider vertebral asymmetries such as non-parallel endplates and concave vertebral walls [26].

Measurements using the Raimondi and Perdriolle methods are more accurate [25]. The intra-observer and inter-observer error for using the Perdriolle method was reported by Weiss to be ±1^o^ and ±3^o^ respectively [25]. Similarly, Barsanti *et al.* and Omeroglu *et al.* reported that over 92% and 98% of errors, respectively, were within ± 5° [27, 28]. However, the accuracy of the measurement reduces when the vertebral rotation is ≥ 30^o^ and the outline of the pedicle loses its definition [5].

Knowing the extent of vertebral rotation is important clinically. When vertebral rotation exceeds 25^o^, trunk rotation increases during forward bending as the paraspinal muscles on both sides of the back act unilaterally to rotate the apical vertebra [29]. This is significant clinically as apical lumbar vertebral rotation in excess of 33% has been shown to be associated with an increased incidence of low back pain [30].

### 2.4 Risser Sign

**Fig. (3):**
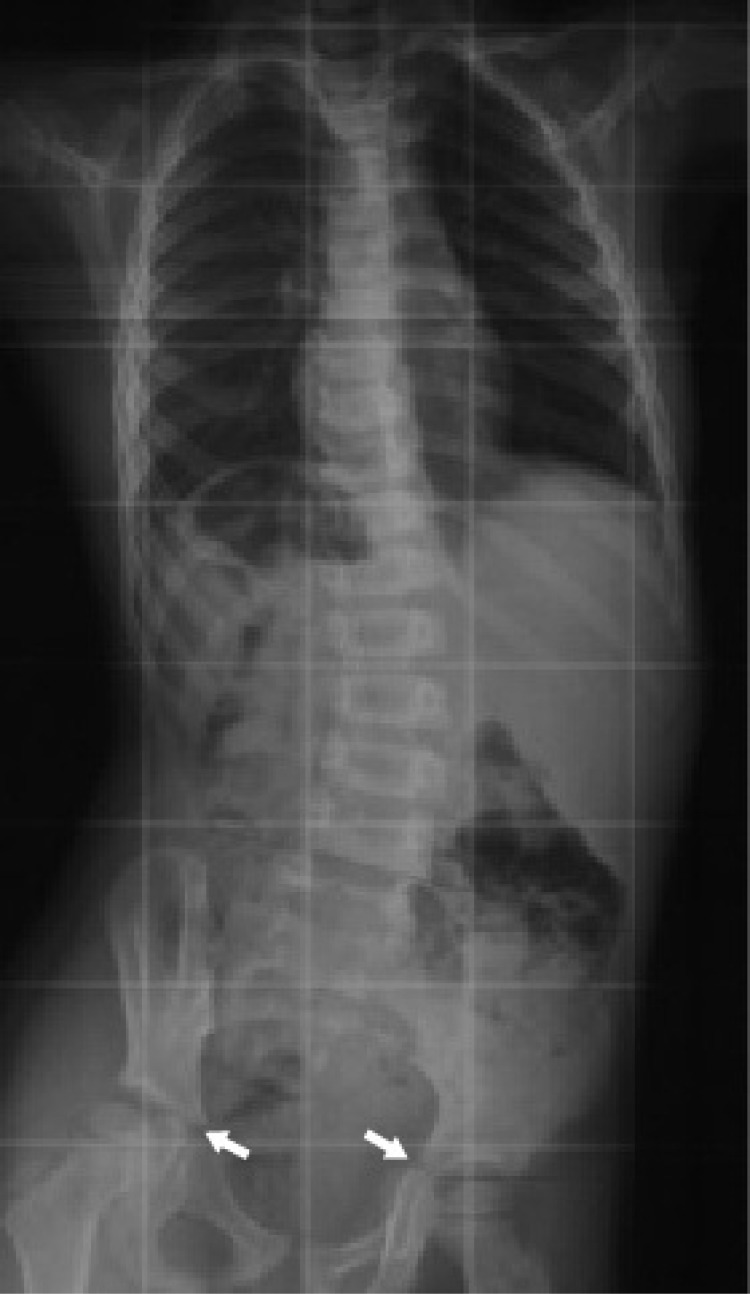
The tri-radiate cartilage. It is the Y shape epiphyseal plate between the ilium (→) ischium and pubis. When it is fused, it indicates that the peak growth velocity phase has passed.

The Risser sign is an important prognostic sign for the management of idiopathic scoliosis. Two different staging systems exist [7, 31], namely the US and French (European) systems. Both systems comprise of 6 stages; they have identical Risser 0 and 5 stages, but different staging in-between (Fig. **3**).

In the US system, the iliac crest is divided into 4 segments. When the iliac apophysis is not ossified, it is regarded as stage 0. When the first lateral quadrant of the apophysis is ossified, it is regarded as Risser 1. With excursion of the ossification medially, the Risser stage increases. In brief, the quadrant the ossification appears within defines the Risser stage [7]. When the second quadrant is ossified, the Risser is 2. When the third quadrant is ossified, the Risser is 3. When the fourth quadrant is ossified, it is Risser 4. When the iliac apophysis fuses with the iliac crest, it is Risser 5 [32].

**Fig. (4):**
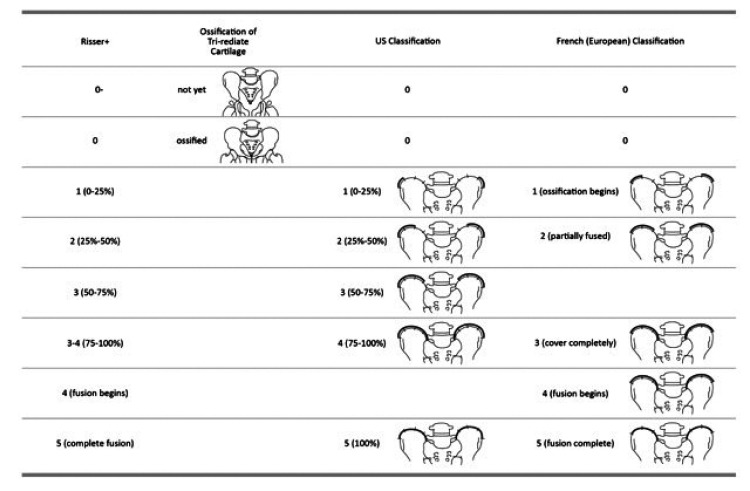
Different classifications of Risser sign. In the Risser + classification, Risser 0- represents Risser 0 stage when the tri-radiate cartilage has not fused, and Risser 0 represents the stage when no ossification of the iliac crest is evident, but the tri-radiate cartilage has fused.

Alternatively, in the European system, the iliac crest is divided into three segments. As with the American classification, in the European classification, when the apophysis ossifies in the outmost segment, it is graded as Risser 1. For a classification of Risser 2, two thirds of the iliac crest needs to have ossified. For a Risser 3, the entire iliac apophysis needs to be ossified. Risser 4 is assigned when the medial iliac apophysis fuses with the ilium [7]. Risser 5 represents the complete fusion of the apophysis to the iliac crest. Recently, Nault *et al.* (2010) and Yang *et al.* (2014) proposed a modification of the existing systems [7, 33]. Nault *et al.* (2010) included the closure of the tri-radiate cartilage into the grading system. When the tri-radiate cartilage has not yet fused Fig. (**3**), it is graded as negative (0 -). When the tri-radiate cartilage has fused, but the Risser sign is still 0, the staging is regarded as 0 [7]. This applies to both the US and European systems (Fig. **4**). Studies have shown that the two systems have high correlation [7, 31].

Prognostically, patients with Risser 0- 2 are regarded as skeletally immature [34] and at high risk of progression. A recent study attempted to determine the influence of Risser sign on the need for surgery in children wearing orthoses for the treatment of AIS [35]. They found that 44.2% of the patients who were in Risser stage 0 and who wore a brace for an average of 11.3 hours per day had curves progress to surgery or to ≥50^o^; 6.9% of patients in Risser stage 1 who wore a brace for an average of 13.4 hours per day progressed to surgery or to ≥50^o^. No patients in Risser stage 2 who wore a brace for an average of 14.2 hours had curve progression to surgery or to ≥50^o^ [35].

Interestingly, Ryan *et al.* (2007) compared the curve progression rates of two groups of patients at Risser 0, one with tri-radiate cartilage open and another with the cartilage fused [34]. They found that the incidence of curve progression is higher in patients with open tri-radiate cartilage as opposed to those with closed tri-radiate cartilages [34]. Despite bracing, patients with an open tri-radiate cartilage progressed a mean of 3.12^o^ and those with a closed or fused tri-radiate cartilage progressed a mean of 6.86^o^ [34]. Considering an increase of 5^o^ Cobb as worsening of curve, curves with open tri-radiate cartilage worsened 54% of the time and those with a closed tri-radiate cartilage worsened 21% of the time. The findings concurred with the findings by Karol *et al.* (2016), who showed that the surgical rate of braced patients with open tri-radiate cartilage was higher than those with closed tri-radiate cartilage. The rate of surgery for patients at Risser stage 0 was 32.4% for the 74 patients with closed tri-radiate cartilage and 63% for the 46 patients with open tri-radiate cartilage (p=0.0005) [35].The outcome is possibly related to the difference in growth velocity when the tri-radiate cartilage is open versus when it is fused. When the tri-radiate cartilage is still open, the growth velocity is high; when the cartilage has fused, the growth velocity reduces [34]. Closure of the tri-radiate cartilage occurs after the peak height velocity [8]. In the study by Ryan *et al.* (2007), the average growth velocity for patients with an open tri-radiate cartilage was 0.34 cm/month and for patients with a closed tri-radiate cartilage the average was 0.23 cm/month. The high growth velocity in Risser stage 0- may contribute to curve progression. Once the tri-radiate cartilage has fused and the patient has passed their peak height velocity, he/she should be regarded as relatively mature [34]. Based on the study outcome (Ryan *et al.* 2007), patients in Risser stages 0-2 who were previously regarded as immature should be regarded more like mature patients.

**Fig. (5):**
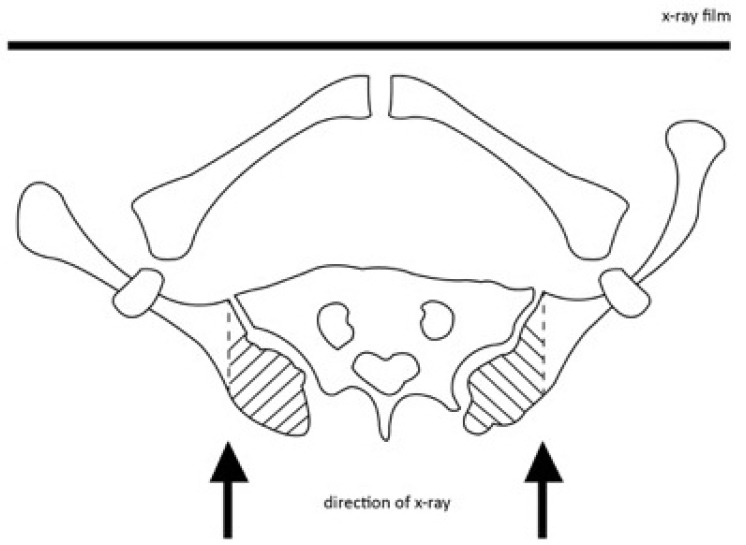
The posterior aspect of the iliac crest (hatched area) is situated behind the sacroiliac joint and is thus obscured by the joint in frontal view projection.

When the U.S. Risser sign reaches 2, the risk of curve progression reduces. Karol *et al.* (2016) showed that no patients managed with bracing for AIS at U.S. Risser 2 had progression to surgery [35]. At Risser 3, it is customary to regard the patient to be relatively skeletally mature. The brace can be weaned off gradually [36], but this has to be managed with care. Determination of Risser 3, 4 and 5 may not be accurate on a frontal film, as the posterior third of the iliac crest orients sagittally and is obscured by the sacroiliac junction (Fig. **5**) [31]. The worst concordance rate for Risser 3 is when comparing a PA against an AP film; Izumi found this to be only 19% [37]. Similarly, Yang *et al.* (2014) showed that the mean concordance rates using plain radiographs and 3D computed tomography were 59.76% and 67.42% using the U.S. and the European grading methods, respectively [33]. Further, skeletal growth and curve progression are possible even after Risser 4-5 [38], Risser values which are usually taken to signify skeletal maturity.

Once a value of Risser 5 has been reached in a patient with AIS, and the patient is generally considered to be skeletally mature, some curves still progress. The incidence of curve progression has been shown to vary from 10% to 68%, depending on the initial curve magnitude [6, 39]. Thus, brace weaning needs to be individualized and should not be based on the Risser stage alone. Weaning should only commence when the curve has been found to stabilize.

It is important to note that Risser sign has high intra-observer and inter-observer reliability [7, 31, 40, 41]. Unfortunately, atypical iliac apophysis development is frequent [32, 33, 39]. This includes fragmented ossification, short excursion of the ossification as well as posterior ossification. The ossified apophysis may also overlap the iliac crest, obscuring the image. It is, however, important to consider that a study by Izumi *et al.* (1995) showed that when the Risser sign of PA and AP films were compared, the agreement was only 58% out of the 89 cases studied [37]. So the measurement of the Risser sign needs to be regarded with caution and in conjunction with other patient measures. Despite these limitations, the Risser sign is still routinely used in the management of scoliosis.

### 2.5 Lateral Spinal Radiograph

A lateral spinal radiograph is generally taken during initial evaluation. In the lateral film, the pelvic incidence, pelvic tilt, sacral angle, the spinal sacral axis, the lumbar lordosis, the thoracolumbar angle (T12-L1) and thoracic kyphosis angle can be determined. A recent study has shown that the apex of primary and compensatory curves is lordotic, with the anterior vertebral measurements longer than that of the posterior [42]. The lordosis does not involve the entire spine, but is limited to the primary and compensatory curves [42].

Schlosser *et al.* (2016) showed that the anterior-posterior difference of spinal length, based on the “true” anterior and posterior points on the vertebral endplates, was +3.8% for thoracic and +9.4% for (thoraco)lumbar curves, while in the proximal and distal junctional segments between the primary and compensatory curves, no clear anterior-posterior vertebral length discrepancy was observed (p <0.001) [42]. The “plus” sign indicated that the anterior vertebral length was longer than that of the posterior. Recently, Schlosser *et al.* (2016) found that the anterior-posterior difference in vertebral length in primary and compensatory curves correlated linearly with Cobb angle and axial vertebral rotation (r>0.729 for thoracic curve; r>0.485 for thoracolumbar curve).

**Fig. (6):**
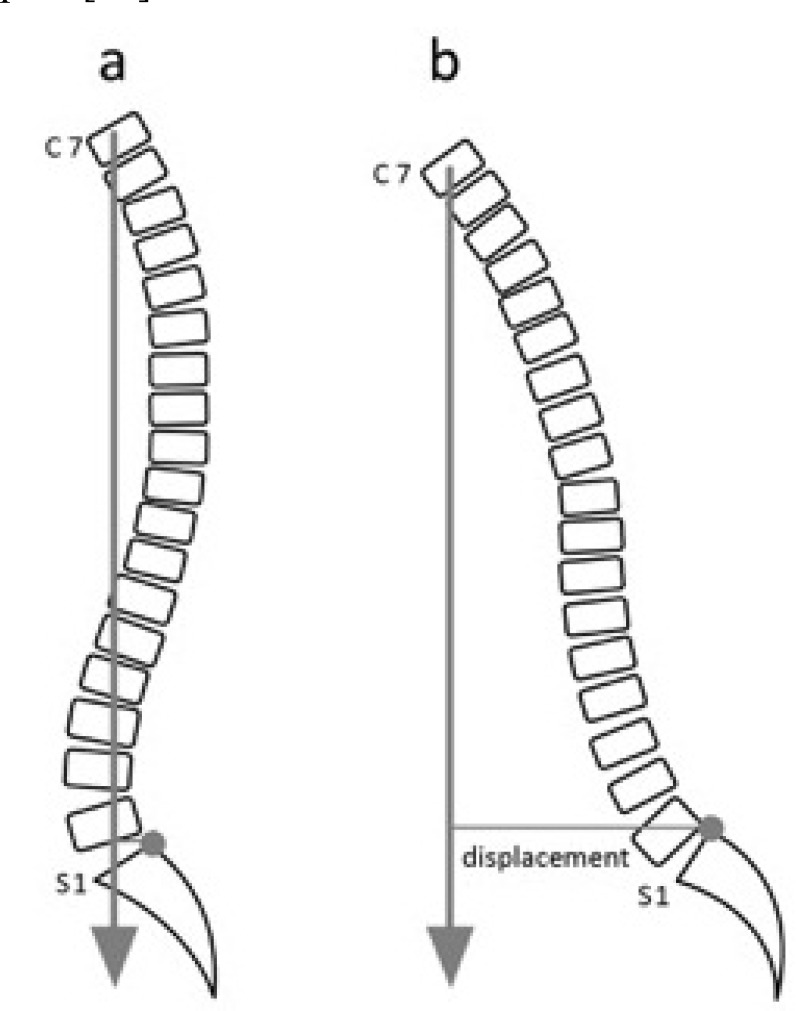
Sagittal imbalance is defined as the anterior displacement of the perpendicular from the body of C7 to the supero-posterior angle of S1. (a) The spine is sagittally balanced. (b) The C7 is anteriorly displaced in relation to S1. This indicates sagittal imbalance.

In adult AIS patients, the sagittal balance is more important than the coronal balance [43]. Positive sagittal imbalance, which is defined as the anterior deviation of the C7 vertebral body plumb line measurement from the supero-posterior corner of the S1 (Fig. **6**) [43], has been found to correlate with adverse health status outcome [44]. Additionally, the presence of a thoracolumbar lordosis together with a positive sagittal imbalance has been found to increase the likelihood of low back pain [44].

**Fig. (7):**
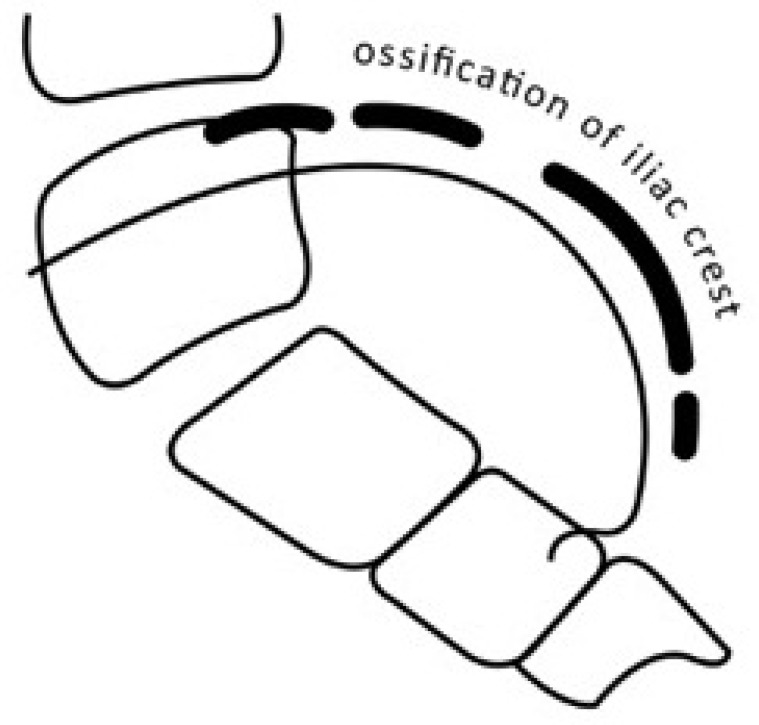
Ossification of the posterior iliac crest is more readily discerned in lateral view of the spine, as it is not obstructed by the sacroiliac joint as in the frontal view.

Kotwicki (2008) demonstrated that lateral spinal radiographs can be used to complement the frontal spinal radiograph in the assessment of the Risser sign [31]. The ossification of the iliac apophysis, particularly the posterior third can be viewed more clearly in the lateral spinal radiograph [31], improving the accuracy of Risser staging. This is especially true for Risser stages 3, 4 and 5 (Fig. **7**).

**Fig. (8):**
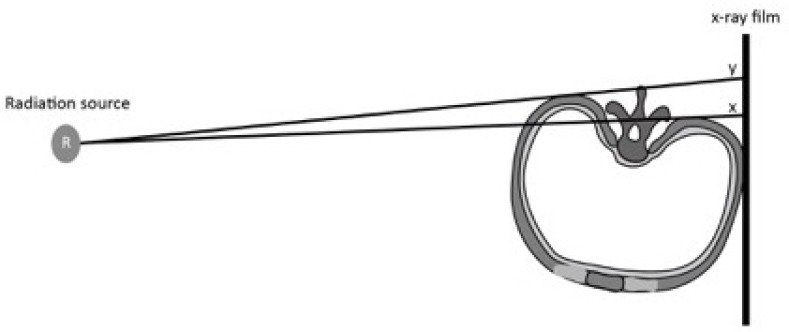
The double rib contour sign. In the presence of truncal rotation, the apical rib on the convex side (y) projects posteriorly in relation to the contralateral rib at the same level (x). This constitutes the double rib contour sign.

In the lateral spinal radiograph, the double rib contour may also be seen [45]. In a normal spine with no rotation in the horizontal plane, images of ribs from both sides should not appear one in front of another in a lateral spinal radiograph. In the presence of scoliosis when there is trunk rotation, the rib contour of the convex side appears posterior as compared to that of the concave side (Fig. **8**). This is the double rib contour sign [45], which is an indirect sign of vertebral rotation.

### 2.6 Frequency of Radiographs

When diagnostic screening of children suggests the possibility of AIS, children should be evaluated by radiography [4]. If no progression is evident, the child should not be radiographed more than once a year. Follow-up radiographs, if needed, should be taken with as few projections as possible [46]. Unless the patient has a marked thoracic hypokyphosis or lumbar hypolordosis, lateral spinal film should not be taken during each examination [3, 9] in order to keep the exposure to radiation as low as possible.

When a brace is indicated, a pre-brace x-ray is required to establish a baseline against which future x-rays are compared [3]. This baseline x-ray also provides the basis upon which the orthotist can design and fabricate the brace. Once the brace has been fitted, an additional in-brace x-ray is required to determine the frontal and lateral balance of the spine together with the degree or percentage of curve correction. This data is essential for the prognosis of the condition [47, 48]. An in-brace correction of 30% or more is required to achieve the best final spinal and rib-cage correction when the patient reaches skeletally maturity [47]. A larger in-brace correction of 40% or more was found [48] to accompany an improvement of 7^o^ Cobb at skeletal maturity. There is, at present, no general consensus for when the in-brace x-ray should be taken [3]. Some physicians routinely take the x-ray on the day of brace fitting. Others suggest to take the x-ray two to four weeks following brace fitting, to allow time for modification of the brace [3]. This allows patients to get used to the brace and also allows the facilitation of viscoelastic changes in the spine through incremental loading of the corrective forces [3].

Similarly, there is no standard protocol relating to when subsequent x-rays should be taken and whether they are to be taken in or out of the brace [3]. Given the lack of consensus, however, it has to be stressed that out-of-brace x-rays need to be taken after the patient has spent some time wearing the brace. This is especially true for infantile and juvenile scoliosis patients, as the corrective forces of the brace may deform the ribs and soft tissues, creating chest wall and/or sagittal plane deformities [3]. Out-of-brace x-rays used to evaluate progress should be taken 24-48 hours out of brace.

Depending on the treatment protocol, the number of radiographs taken during the treatment period necessarily differs. Presciutti *et al.* (2014) reviewed the treatment charts of the idiopathic scoliosis patients they had seen between 2007 -2012 and found that patients treated by observation had a mean of 3.7 radiographs per year, those treated by bracing had 5.7 radiographs per year and those treated by surgery had 12.2 radiographs per year [49]. Although this is significantly less than the 22 radiographs taken in three years reported by Nash *et al.* (1979) [50], 12 radiographs per year is still very high and can increase the risk of breast cancer [49].

### 2.7 Radiation Dose and Hazard

The effects that the radiation have on the tissues or organs depend on three factors: the amount of radiation absorbed, the type of radiation that the patient is exposed to, and the type of tissues that are irradiated. The radiation doses that are actually absorbed by the tissue/s is equivalent to the concentration of energy deposited in the tissue/s as a result of an exposure to ionizing radiation. It is measured in milligrays (mGy). The “equivalent dose” is an amount that takes the damaging properties of different types of radiation into account. It varies with the type of radiation. It is measured in milliSievert (mSv). The relationship is as follows:

Absorbed dose (mGy) x radiation weighing factor = equivalent dose (mSv)

As radiography has a radiation weighting factor of 1, the absorbed dose is the same numerically as the equivalent dose, though with different units.

The effective radiation dose takes into account the sensitivities of tissues and organs to radiation, as different body parts have different sensitivities to radiation. The relationship between equivalent dose and effective dose is as follows:

Equivalent dose (mSv) x tissue weighting factor = effective dose (mSv)

From the equation it can be seen although both the equivalent and effective doses are in mSv units, they refer to different radiation doses.

It is important to consider that radiography is associated with ionizing radiation, which has been found to increase the risk of malignancy [50-52]. Many studies have shown that AIS patients are at increased risk of having breast cancer from repeated radiographic examination [49-51]. Simony *et al.* (2016) investigated the cancer rate of 170 AIS patients treated between 1983 and 1990 [52]. On average, each patient had 16 radiographs throughout the entire treatment. The calculated mean radiation exposure was 0.8 – 1.4 mSv per examination and 2.4 – 5.6 mSv/year. This radiation dose was comparable to that generated by modern radiography equipment [52]. Of the studied cohort, 9 patients were found to have cancers. Four had endometrial cancer and three had breast cancer. The overall cancer rate was 4.3%, which was five times higher than the age-matched Danish population [52]. Interestingly, the authors found that patients who had endometrial cancer were treated at an earlier age than those who had breast cancer [52]. The reason for this discrepancy is unknown but could possibly be related to the response of different biologic tissues or organs to radiation based on the patients’ age or physiological maturity [52].

Additionally, Presciutti *et al.* (2014) compared the radiation dose received by patients who were treated by observation, bracing and surgery. They found that the surgical group received 8-14 times more radiation than the braced and observation groups [49]. These results suggest that it is imperative to find ways to reduce the radiation dose to patients destined for surgery.

### 2.8 Reducing the Radiation Dose

As discussed above, reducing the radiation dose to patients may help reduce the risk of cancer [53]. This could be achieved through a number of methods: viz. reducing the number of radiographs to as few as possible [54], reducing the exposed area [9] and using the optimal radiographic technique.

Houghton *et al.* (1986) suggested using X-rays (PA) for patients with AIS on three occasions only [55]: At the initial visit to confirm the diagnosis, when there has been rapid growth or change in the deformity and before surgery to locate the exact levels for spinal fusion [56].

However, a lateral spinal radiograph should only be taken during the initial assessment of a scoliosis patient. It should be not taken together with a PA film on every subsequent x-ray examination [3] unless the patient has a significant sagittal plane deformity. For the frontal film, a PA projection is preferable to AP film, despite the fact that an AP film produces a better image quality than a PA film, as it substantially reduces the patient’s effective dose [57, 58] due to the fact that the focal film distance is shorter.

**Fig. (9):**
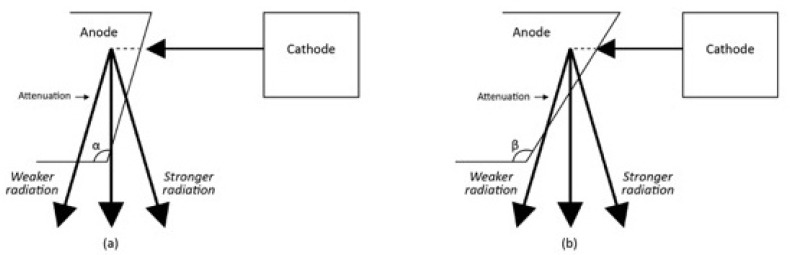
The anode heel effect. The strength of the radiation is weaker in anode as compared to the cathode. The effect stems from the absorption of the X-ray photons as they leave the anode. The effect varies with the heel angle of the anode (α, β). In an anode with a smaller heel angle (α), the X-ray photons have to traverse a longer distance to exit the anode. This attenuates the X-ray photons more. By contrast, in an anode with a larger heel angle (β), the X-ray photons have to traverse a shorter distance to exit the anode. The radiation is thus stronger. In either instance, however, the radiation towards the anode is less than that towards the cathode.

The effective radiation dose to the patient is also influenced by the positioning of the X-Ray’s anode or cathode in relation to body part, or the anode heel effect phenomenon [58]. In general, the x-ray beam consists of a central ray and a divergent beam. The rays that are parallel or near parallel to the inclined anode get absorbed by the anode itself (Fig. **9**). The x-ray intensity at the anode is therefore lower than that on the cathode. Thus taking scoliosis radiography with the patient’s head towards the anode versus towards the cathode changes the patient’s effective dose [58]. It was found that when the head was positioned towards the anode (Head Towards Anode HTA) position this was associated with a smaller effective dose to the patient as compared to the head towards the cathode (Head Towards Cathode HTC) position. With HTA positioning, a right lateral projection reduced the effective dose to 85% and 84% when compared with a left lateral projection for patients aged between 10 and 15 years. An AP-HTA projection caused a 183% and a 181% larger effective dose than a PA-HTA and breast-absorbed-doses in excess of 555% and 879% for patients aged between 10 and 15 years [58]. Thus if possible, the patient should be radiographed in a right lateral and PA position to reduce the effective radiation dose. A PA film reduces both the risk of breast [50, 59] and thyroid cancer for scoliosis patients exposed to repeated radiographs.

Further, initial radiographs should include the pelvis to assist in the diagnosis of the patient. The initial radiograph should also include Risser staging. Subsequent full spinal x-rays can be limited to the exposure of the areas of interest, viz. from C7 to S3 [9]. The cervical spine, ribs and lower pelvis can be excluded to reduce radiation exposure [9] and x-ray shielding of genitals needs to be employed.

## 3 EOS^®^

EOS^®^ is a low dose radiographic equipment that uses an ultra-sensitive multi-wire proportional chamber detector to detect x-rays. The system simultaneously takes the AP and lateral images of the whole body in a standing position in a calibrated environment. The 2D images that are taken can then be reconstructed to form 3D images using dedicated software.

EOS^®^ has a low radiation dose with an image quality of the spine that is comparable to that of computed radiographs [60]. As EOS^®^ enables 3D reconstruction, it enables both the measurement of 3D angles as well as the measurement of distances [61]. This usually requires imaging by computed tomography that has a higher radiation dose. Moreover, studies have shown that 3D measurements from the EOS^®^ has very high intra- observer repeatability for the Cobb angle, thoracic kyphosis and lumbar lordosis with better inter-observer reproducibility than the 2D methods [62]. The measurement of AVR was also very accurate; the average apical vertebral orientation as measured by EOS® and CT scanning was 9.31^o^ and 6.61^o^ respectively (p = 0.65) [63]. The positioning of the patient did not affect the measurements significantly. Additionally, and most importantly, any malpositioning of patients to ±10^o^ did not significantly impact the accuracy of the EOS® reconstructed images [63, 64].

Recently, an EOS^®^ microdose protocol was introduced to further reduce the radiation exposure to patients, particularly to pediatric patients who require repeated radiographs [65]. Ilharreborde *et al.* (2016) evaluated the precision of the 3D reconstruction of radiographs using this new microdose protocol. They found that the reconstructed images were accurate. The intra-operator repeatability was better than the inter-operator reproducibility for all parameters with values ranging between 3^o^ and 8^o^ for frontal and sagittal parameters and between 1^o^ and 8^o^ for pelvic measurements [65]. The agreements were good for all measurements (ICC >0.91) [65], suggesting that the microdose protocol can be clinically used for the monitoring of scoliosis.

### 3.1 Radiation Dose

EOS^®^ has been in clinical use since 2000. It reduces radiation exposure by approximately 6 times that of conventional radiography [54, 60, 63, 65]. Recently, as a result of technical advances, the microdose protocol was developed further reducing the radiation dose to patients.

In conclusion, the radiation dose of low dose EOS^®^ is significantly lower than that of conventional radiography. For a frontal film, the radiation dose with low dose EOS^®^ was 0.07mGy as compared to 0.92mGy for a conventional radiograph [66, 67]. For a microdose protocol, the radiation dose is even lower and is around 5.5 fold less than that of the low dose EOS^®^ [65]. This translates to a 45 fold reduction in radiation compared to conventional radiographs, making the radiation dose to the patient negligible (Table **1**) [65].

## 4 SURFACE TOPOGRAPHY (RASTERSTEREOGRAPHY RS)

Repeated radiographic examination over time is generally required in AIS patients to document the treatment effectiveness or progression of the deformity. These serial radiographs may result in relatively high cumulative radiation doses. It is from this background that surface topography was developed [68], with the objective of reducing the need for radiography in the monitoring and management of scoliosis. At present, many surface topography systems are in use. The common ones include the Jenoptik Formetric system [69], Inspeck System [70], Integrated Shape Imaging System version 2 (ISIS2) [71] as well as the Quantec Spinal Imaging System [72]. Current data supports the accuracy and reproducibility of all four systems [69].

The underlying principles of the different systems are similar. The devices project structured or stripes of white light (raster lines) onto the back of a patient, either sequentially or simultaneously. Multi-light sectioning refers to the technique when all the raster lines are projected onto the back of the patient at the same time. The latter method is preferable as the time to capture a frame is shorter, reducing the possibility of blurring of images due to patient movement. Deformed projected light on the back of the patient is then recorded by a video camera. In combination with calibration data, 3-D images of the sectioned surface points are constructed using the triangulation method [61].

The preparation of patients differs, depending on the systems. Some require marking bony landmarks with external skin markers [71], while others [61] detect the bony landmarks automatically. The latter method reduces the need for an experienced operator [71] and error due to palpation [61].

Weiss *et al.* showed that some trunk surface parameters altered significantly during maximum inspiration as compared to end expiration [74]. They compared the surface measurements of 77 AIS patients with a mean Cobb angle of 38^o^ using Formetric device in maximal inspiration and end expiratory positions. They found that the kyphotic angle (p<0.001), lordotic angle and the spinal length differed significantly in the two positions [73]. To avoid breathing artefacts, they recommended measuring trunk surface parameters at the end of expiration [74].

### 4.1 Reliability of Rasterstereography

A number of studies have attempted to determine the reliability of the surface topography measurements as compared to conventional radiography [69, 74, 75]. A recent systematic review of twelve studies evaluating the validity of RS shows that the accuracy of the method varies [76]. RS cannot accurately determine Cobb angle, though the measurements correlate well with that measured on x-rays [69, 74, 75, 77]. A recent multicenter study found that the 4D Formetric underestimated Cobb angle by a mean of 8.12 degrees [69]. Similarly, Bettany *et al.* found that ISIS1 underestimated large curves and overestimated small curves [56]. They found that the correlation with Cobb angle was best for thoracic and thoracolumbar curves [56]. Correlation was also high for the double and primary lumbar curves. The poorest correlation was found for the compensatory lumbar curves [56]. The best correlation was found for medium curves in the 25-40^o^ range [56].This is understandable in view of the fact that RS measures the surface of the back whereas x-rays measure the internal skeletal elements of the spine [56].

Weiss *et al.* (1997) determined the margin of error for the measurement of lateral deviation and rotation using the Formetric system [73]. They reported that the errors of maximum and average lateral deviation were 6mm and 3 mm respectively and that of maximum and average trunk rotation to be 3^o^ and 1^o^, respectively. The lateral deviation [79] and trunk rotation [80, 81] correlated better with that determined by radiography.

Liljenqvist *et al.* reported an unacceptable high root mean square (rms) difference for a vertebral rotation of 7.9 degrees comparing RS and radiography. However, other authors [78, 79, 82] reported a smaller rms difference comparing digitized x-rays and RS [76]. Schulte *et al.* in a longitudinal study averaging 8 years showed that the progression of lateral deviation and vertebral rotation of curves closely correlated with those obtained by radiography [79]. The mean difference between RS and radiographs was 3.21mm for lateral vertebral deviation and 2.45^o^ for vertebral rotation [79].

The reliability of RS in determining the sagittal profile has not been studied extensively [61, 76]. Weiss *et al.* compared the absolute values of thoracic kyphosis as measured in RS and in radiographs [83]. They found a correlation of 0.78 between the two values, with a mean difference of 14 degrees. Yet, it has to be noted that the radiographic thoracic kyphotic angle was measured from T4 to T12, whereas the RS kyphotic angle was measured from T1 to T12 [76]. This means, in essence, that different angles were being compared by the two different instruments. Similarly, Knott *et al.* found that the Formetric 4D back shape measuring system overestimated the thoracic kyphosis by an average of 7.26 degrees [69].

**Fig. (10):**
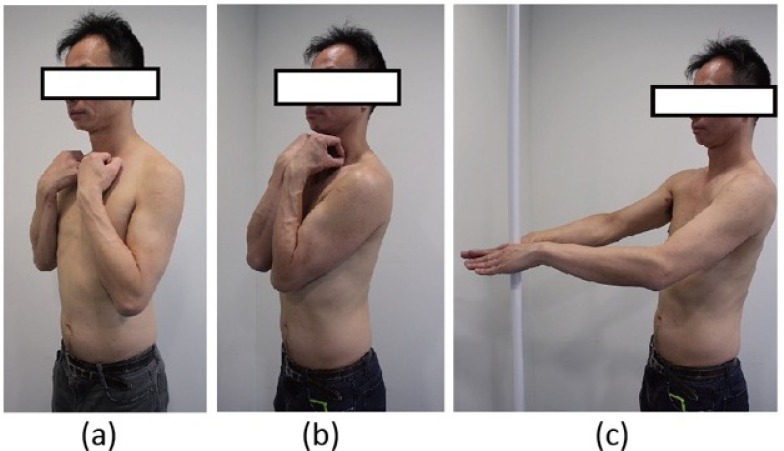
The different standing positions used in the measurement of trunk surface asymmetries using surface topography. (a) The clavicle position; (b) the folding position and (c) the straight out position.

In order to improve the reliability of RS for the measurement of sagittal alignment, de Sèze *et al.* compared the measurements taken in three different standing positions (Fig. **10**), *viz*. the clavicle position, the folding position and the straight out posture [84]. They found that the straight out posture, *i.e.* standing with arms and hands supported horizontally in front is closest to the natural standing posture. Parameters evaluating the upper part of the trunk presented higher reproducibility than the other two postures [77].

### 4.2 Clinical Applications

As the RS data correlates well with the spinal deformity, RS can be used for screening and monitoring scoliosis [69]. While RS cannot replace radiography entirely, it can reduce the need for x-rays. Radiographs can be reserved for cases when progression of curves is documented or surgery is planned. Also, RS may be useful in monitoring curve progression and posture in pregnant women when radiography is contraindicated [85, 86].

The use of RS in screening scoliosis is controversial [87, 88]. Chowanska *et al.* investigated if RS could replace the scoliometer in screening for scoliosis, using a portable rasterstereography device (CQ Electronic System, Wroclaw, Poland), and concluded that RS is not suitable for scoliosis screening. The sensitivity and specificity of the imaging method were not satisfactory. For the value of surface trunk rotation (STR) ≥ 5°, the sensitivity was 64.5% and the specificity was 88%. For the value of STR ≥ 4° the sensitivity was 77.4% and the specificity was 71.1%. No STR value simultaneously provided a satisfactory sensitivity and a satisfactory specificity [87]. The cut-off value for the STR parameters to determine if the patient had scoliosis or not could not be established. Also, the screening process took longer and the children had to uncover the entire back [87]. Conversely, using an experimental RS setup, Pino-Almero *et al.* reported that RS has a higher specificity and sensitivity than the scoliometer in detecting scoliosis and recommended its use in the detection of scoliosis to reduce the use of radiography [88]. The difference in the interpretation of the results is possibly related to the fact that the former study considered the practical application of the screening while the latter study only considered the technical aspect of the findings.

Further, RS has been shown to be effective in monitoring the progression of curves [74, 79, 89, 90]. Schulte *et al.* (2008), in a longitudinal study, found that an increase in lateral deviation and trunk rotation correlated well with the progression of the curves and suggested that RS can be used reliably to monitor the progression of curves [79]. Similarly, de Korvin *et al.* (2014) reported that an increase of ≥ 2^o^ in any one gibbosity or in the sum of the gibbosities in an RS examination indicated a 5^o^ increase in Cobb angle [89]. Theologis *et al.* (1997) using ISIS also showed that rib hump may progress before the Cobb angle [91]. RS is thus able to specifically indicate the progression of curves and reduce the number of radiographs required for follow-up [79, 89] up to 50% [89]. Schulte *et al.* (2008) recommended an RS examination every 3 to 6 months and a radiographic examination every 12 to 18 months, provided that the RS examination does not show rapid deterioration of the scoliosis [79]. Surface topography may also be used to assess changes in the back surface after surgery [92].

## 5 3D ULTRASOUND

Similar to surface topography, 3D-ultrasound has been developed in an attempt to reduce radiation exposure to AIS patients. Letts *et al.* were the first to use ultrasound to assess scoliosis curves [93]. They applied ultrasonic digitization to identify spinous processes and documented spinal curvature using the Ferguson method [93]. In the past decade, there have been an increasing number of studies investigating the possibility of using ultrasound images to determine spinal curvatures [94-96], vertebral rotation [97-99] and Risser sign [100-102].

Ungi *et al.* used the ultrasound system with a tracked transducer to determine spinal curves [94]. They used transverse processes as landmarks. Results showed that the Cobb angle determined using the ultrasound imaging method correlated very well with that determined using radiographs. It has to be noted, however, that the data was obtained from pediatric and adult phantoms.

Suzuki *et al.* and Chen *et al.* used spinous process and vertebral lamina as bony landmarks [97, 103]. Suzuki *et al.* used ultrasound imaging to measure vertebral rotation in AIS patients in the prone position. They used the spinous process and lamina as landmarks to calculate vertebral rotation and found that there was a strong linear relationship between vertebral rotation and Cobb angle in thoracic and lumbar curves in untreated AIS patients [97]. When the patients were braced, however, the relationship was lost. Chen *et al.* (2011) proposed to use the centre of lamina method (COL) to estimate the curvature and vertebral rotation of scoliosis. The method has subsequently been used [95, 99] in a number of studies.

Wang *et al.* attempted to validate the accuracy and reliability of the measurement of Cobb angle and vertebral rotation with the 3D ultrasound [95, 99]. They scanned the spine of the patient lying supine on a purpose-design couch with a central rectangular slot [95]. Positioning of the patient was similar to that of MRI scanning in routine clinical examination [95]. They identified the apical vertebra and the lamina of the upper and lower end vertebrae from the ultrasound image manually and used dedicated software to calculate the Cobb angle [95] and vertebral rotation [99]. They found that the measurements correlated very well with that determined from MRI taken a few hours prior to the ultrasound imaging [99]. The ultrasound scanning method was regarded as having a very good intra- and inter-rater reliability [95, 99, 104], but it under-estimated the Cobb angle when compared to that determined by radiography [104]. In an attempt to improve the accuracy of the ultrasound imaging, Zheng R *et al.* used previous radiographs of patients to aid identification of bony landmarks and measurement of Cobb angles. They found that the ultrasound imaging, with the aid of previous radiographs improves the accuracy and reliability of the measurement of the Cobb angle on patients with AIS [96].

Ultrasound imaging has also been used to evaluate Risser sign staging. Between Risser 1 and 3 stages, the correlation of ultrasound imaging with radiography was high [100-102], but this was not found for Risser 4 and 5 [102]. The inconsistencies in identification of Risser stages 4 and 5 by ultrasonography may be due to the overlap of the images by the transverse process of L5 and that of the medial iliac apophysis [101]. Patients with Risser stage 4, with the medial iliac apophysis not yet completely ossified may be regarded as Risser stage 5, as the bony structure of the transverse process of L5 may be mistakenly regarded as ossification of the medial iliac apophysis [101].

### 5.1 Limitations of 3D Ultrasound

The method, though promising is not without limitation. Part of the limitation is intrinsic to the technology while other factors are extrinsic, relating to the curve severity, curve type and the morphology of the patient [104]. The flat transducer of the ultrasound machine did not entirely make full contact with the skin in some patients, particularly in those with large humps [99] and with winged scapulae [104]. This resulted in some missing images and difficulties in identification of bony landmarks.

Also, the image of the spine was poor in those with high BMI (25 kg/m^2^) [104]. This is possibly related to the attenuation of the ultrasound signals by the thick subcutaneous tissues. Obesity also increases the difficulty of the assessment of the Risser sign [100, 102]. Torlak *et al.* (2012) showed that the evaluation of the iliac apophysis was more difficult with a subcutaneous fat thickness in excess of 16 mm. For patients with a Cobb angle in excess of 50^o^, the image was poor as well [104]. This was due to the marked vertebral rotation of the spine [104]. Similar to surface topography, the Cobb angle measured is smaller than that made from radiographs [104]. This may be related to the fact that ultrasound images are taken posteriorly and provide anatomical features of vertebral posterior elements rather than the vertebral bodies used in Cobb angle measurement from radiographs [105].

## 6 COMPUTED TOMOGRAPHY

Computed tomography is another imaging method that provides excellent visualization of the bony spinal column and permits the accurate measurement of vertebral rotation. Despite this, it is not used in the monitoring of scoliosis as the radiation dose is high. The main role of CT is to assess for any underlying occult pathology and for the pre-operative planning of scoliosis. There are currently no clear guidelines on which children with scoliosis should have the more advanced (and expensive) imaging methods [12].

Additionally, in patients with suspected osteopenia, dual-energy x-ray absorptiometry (DEXA) may be indicated. It uses a very small dose of ionizing radiation to obtain images of the lumbar spine and hips to measure bone density. The Cobb angle was found to be inversely and independently related to the bone mineral density and bone mineral content in peripubertal girls [106]. Osteopenia has also been associated with a higher risk of curve progression [107].

## 7 MAGNETIC RESONANCE IMAGING (MRI)

When the clinical history and physical examination elicit certain worrying features such as pain, neurological symptoms or an atypical curve pattern, patients should be referred for advanced imaging. This allows for early and accurate detection of an underlying cause, allows for optimal planning and timing of surgery and helps reduce associated risks. The presence of congenital bony defects should also prompt MRI examination.

When radiographs show evidence of congenital defects, including defects of formation, such as hemi-vertebrae, anteriorly wedged and butterfly vertebrae and defects of segmentation such as unsegmented bars and block vertebrae, the patient should be referred for MRI examination as the defects are associated with a high incidence of spinal cord anomalies [11-14, 108, 109]. Trenga *et al.* (2016) reported 55% of the 75 patients with congenital defects had associated spinal cord anomalies. Patients with a mixed formation and segmentation defects had a higher incidence of spinal cord anomalies than those with only formation or segmentation defects alone. Also, the incidence of spinal cord anomalies was higher when two or more vertebrae were involved [109]. The rate of spinal cord anomalies (87%) was highest when the deformity involved the sacroccygeal area [109]. Of interest is that MRI revealed 47% of the bony anomalies not seen in radiographs, as it overcame the obscuring effects of the skull and pelvic viscera and improved the characterization of the abnormal vertebrae [109].

The prevalence of spinal cord abnormalities in infantile and juvenile “presumed” idiopathic scoliosis ranges from 11.1% to 26.0% [15]. Zhang *et al.* (2016) examined 504 infantile and juvenile patients diagnosed with “presumed idiopathic” scoliosis by MRI for potential neural axis abnormalities. The patients were all below the age of 10 at diagnosis, had an initial primary curve of >20^o^ and normal neurological findings on history and physical examination. Results showed that 18.7% of patients had a neural axis abnormality. In total, Arnold-Chiari malformation with or without syringomyelia accounted for 64.8% (61/94) among all the abnormalities [15]. The study found that male gender, left thoracic curve and right lumbar curve are significantly associated with higher incidence of neural axis abnormalities [15]. It is therefore very important that patients with infantile and juvenile scoliosis with an atypical scoliosis pattern have MRI examination.

The incidence of neuraxis abnormalities in patients with AIS is lower than that of JIS; it is reported to range from 3% to 4% [110, 111]. AIS patients with atypical scoliosis curve patterns, which include left thoracic curve, short curve (4-6 segments), reduced vertebral rotation, absence of apical vertebral lordosis and rapidly progressing curves [112] or neurological signs should be referred for MRI examination to rule out the possibility of neuraxis abnormalities.

## CONCLUSION

In summary, different imaging techniques have different indications and applications in the management of AIS. Radiography is currently the gold standard in the evaluation and management of scoliosis curves. The current consensus is to reduce radiographs to as few as reasonably possible and to reduce the radiographic exposure to just the region of interest. EOS^®^ is at present an ideal imaging method for evaluating scoliosis. Yet, it is very expensive and is not commonly available. Surface topography is very useful in documenting the changes in body contour and can supplement the use of radiography, thus reducing the radiation dose to the patient. 3D ultrasound scanning is at present at an early stage of development and still has a number of limitations. Finally, computed tomography is playing a diminishing role in the management of spinal deformity and is gradually being replaced by MRI especially in the diagnosis and pre-operative planning of scoliosis.

## Figures and Tables

**Table 1:** Radiation exposure (in mGy) of different imaging techniques for spinal examination (Richards *et al.* 2010).

-	Full spine film	
Imaging Techniques	Frontal (mGy)	Lateral (mGy)
EOS^®^ microdose	0.019	0.044
EOS^®^ low dose	0.132	0.214
Conventional Radiography	1.662	1.862
Full spine CT scan	15.6	-
Low dose full spine CT scan	5	-

CT – computed tomography
